# 400. Impact of SARS-CoV-2 Test-based Strategy to Reduce Quarantine Days Among Asymptomatic Healthcare Workers After Household Exposure

**DOI:** 10.1093/ofid/ofab466.601

**Published:** 2021-12-04

**Authors:** Bhagyashri D Navalkele, Jose Lucar, James B Brock, Jason Parham

**Affiliations:** University of Mississippi Medical Center, Jackson, MS

## Abstract

**Background:**

Appropriate staffing is essential to provide safe patient care. During the COVID-19 pandemic, healthcare workers (HCWs) are missing work days due to illness or high-risk exposure (HRE) to an infected person. To avoid staffing shortages, we implemented a SARS-CoV-2 test-based strategy among asymptomatic HCWs after HRE to facilitate early return to work.

**Methods:**

In July 2020, our institution implemented a SARS-CoV-2 RT-PCR test-based strategy among HCWs within 7 days of HRE. HCWs were defined as any paid or unpaid persons directly or indirectly involved in patient care. HRE was defined as close contact < 6 feet with an infected household member without use of mask and lasting for ≥ 15 minutes. Contact with a patient or coworker was not considered high-risk due to universal masking and eye protection use. HCWs underwent SARS-CoV-2 RT PCR testing of a nasopharyngeal swab at least once (1-2 days post-exposure) or twice (5-7 days post-exposure). HCWs with symptoms at baseline were excluded. HCWs who were asymptomatic during evaluation were considered as truly asymptomatic (TA). Saved work-days (SWD) were calculated based on number of days saved due to testing strategy compared to the Centers for Disease Control and Prevention’s recommended 14-day quarantine. HCWs were allowed to return to work within 7 days of HRE if they tested negative, or after completing 10-day isolation period ± improvement in symptoms from symptom onset if they tested positive.

**Results:**

Between 07/01/2020 to 12/31/2020, 450 unique asymptomatic HCWs underwent SARS-CoV-2 testing. Of those, 84% were women and median age was 36 years, 347 tested negative and 103 tested positive. Of those positives, 33% of HCWs tested positive on day 2 after HRE with 141 SWDs (average 2 days/person). Only 37% were TA positives. Of those negatives, 94% were TA SARS-CoV-2 negative with 2620 SWDs (average 7.5 days/person). There were no healthcare outbreaks related to HCWs allowed to return to work following this strategy.

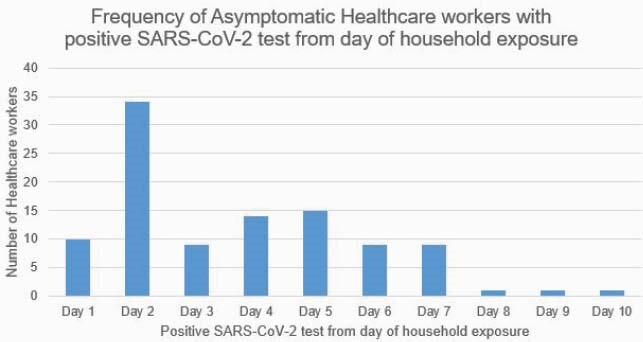

Asymptomatic healthcare workers commonly tested positive for SARS-CoV-2 on day 2 from household exposure compared to other days

**Conclusion:**

Test-based strategy among asymptomatic HCWs with HRE reduced loss of workdays and helped limit staffing shortages. Majority of positive HCWs developed symptoms after positive SARS-CoV-2 testing, which may support allowing most fully vaccinated HCWs with no COVID-like symptoms to continue to work unless symptomatic.

**Disclosures:**

**All Authors**: No reported disclosures

